# Specific detection of methionine 27 mutation in histone 3 variants (H3K27M) in fixed tissue from high-grade astrocytomas

**DOI:** 10.1007/s00401-014-1337-4

**Published:** 2014-09-09

**Authors:** Denise Bechet, Gerrit G. H. Gielen, Andrey Korshunov, Stefan M. Pfister, Caterina Rousso, Damien Faury, Pierre-Olivier Fiset, Naciba Benlimane, Peter W. Lewis, Chao Lu, C. David Allis, Mark W. Kieran, Keith L. Ligon, Torsten Pietsch, Benjamin Ellezam, Steffen Albrecht, Nada Jabado

**Affiliations:** 1 Departments of Experimental Medicine and of Human Genetics, McGill University, 4060 Ste Catherine West, PT239, Montreal, QC H3Z2Z3 Canada; 2 Institute of Neuropathology, University of Bonn Medical Center, Sigmund-Freud-Strasse 25, 53127 Bonn, Germany; 3 Clinical Cooperation Unit Neuropathology, German Cancer Research Center, Heidelberg, Germany; 4 Division of Pediatric Neurooncology, German Cancer Research Center (DKFZ), Heidelberg, Germany; 5 Department of Pediatrics, Montreal Children’s Hospital, McGill University Health Centre, Montreal, QC Canada; 6 Department of Pathology, Montreal Children’s Hospital, McGill University Health Centre, Montreal, QC Canada; 7 Research Pathology Facility, MCETC, Jewish General Hospital, Montreal, QC Canada; 8 Department of Biomolecular Chemistry, School of Medicine and Public Health, University of Wisconsin, Madison, WI USA; 9 Laboratory of Chromatin Biology and Epigenetics, The Rockefeller University, New York, NY USA; 10 Division of Pediatric Hematology/Oncology, Department of Pediatric Oncology, Dana-Farber Cancer Institute, Boston Children’s Hospital, Harvard Medical School, Boston, MA USA; 11 Center for Molecular Oncologic Pathology, Dana-Farber Cancer Institute, Harvard University, Boston, MA USA; 12 Department of Pathology, CHU Ste-Justine, Université de Montréal, Montreal, QC Canada; 13 Montreal Children’s Hospital, Montreal, QC H3H1P3 Canada; 14 Hopital Ste Justine, Montreal, QC Canada

**Keywords:** K27M, Histone 3 variants, IHC, K27 trimethylation, High-grade astrocytomas

## Abstract

Studies in pediatric high-grade astrocytomas (HGA) by our group and others have uncovered recurrent somatic mutations affecting highly conserved residues in histone 3 (H3) variants. One of these mutations leads to analogous p.Lys27Met (K27M) mutations in both H3.3 and H3.1 variants, is associated with rapid fatal outcome, and occurs specifically in HGA of the midline in children and young adults. This includes diffuse intrinsic pontine gliomas (80 %) and thalamic or spinal HGA (>90 %), which are surgically challenging locations with often limited tumor material available and critical need for specific histopathological markers. Here, we analyzed formalin-fixed paraffin-embedded tissues from 143 pediatric HGA and 297 other primary brain tumors or normal brain. Immunohistochemical staining for H3K27M was compared to tumor genotype, and also compared to H3 tri-methylated lysine 27 (H3K27me3) staining, previously shown to be drastically decreased in samples carrying this mutation. There was a 100 % concordance between genotype and immunohistochemical analysis of H3K27M in tumor samples. Mutant H3K27M was expressed in the majority of tumor cells, indicating limited intra-tumor heterogeneity for this specific mutation within the limits of our dataset. Both H3.1 and H3.3K27M mutants were recognized by this antibody while non-neoplastic elements, such as endothelial and vascular smooth muscle cells or lymphocytes, did not stain. H3K27me3 immunoreactivity was largely mutually exclusive with H3K27M positivity. These results demonstrate that mutant H3K27M can be specifically identified with high specificity and sensitivity using an H3K27M antibody and immunohistochemistry. Use of this antibody in the clinical setting will prove very useful for diagnosis, especially in the context of small biopsies in challenging midline tumors and will help orient care in the context of the extremely poor prognosis associated with this mutation.

**Electronic supplementary material:**

The online version of this article (doi:10.1007/s00401-014-1337-4) contains supplementary material, which is available to authorized users.

## Introduction

Recent years have seen an explosion of genomic data across many types of human cancer including high-grade gliomas, through the increasing use of next generation sequencing (NGS) technologies and efforts of independent laboratories, as well as large consortia such as The Cancer Genome Atlas (TCGA) and the International Cancer Genome Consortium (ICGC). In children and young adults, critical epigenetic alterations were discovered in subgroups of these tumors. Somatic recurrent mutations resulting in amino acid substitutions at lysine 27 (p.Lys27Met, K27M) and glycine 34 (p.Gly34Arg/Val, G34R/V) in *H3F3A*, which encodes histone 3 (H3) variant 3 (H3.3), were shown by our group and others to characterize about one-third of pediatric high-grade astrocytomas (HGA) [20, 24]. These mutations resulted in somatic heterozygous lysine 27 to methionine (p.Lys27Met, K27M) and glycine 34 to arginine or valine (p.Gly34Arg/Val, G34R/V) amino acid substitutions at these positions within the H3.3 tail [20, 24]. A large cohort of 784 gliomas of all ages and histopathological grades (WHO I–IV) confirmed the prevalence of *H3F3A* mutations in pediatric and young adult HGA [20] as did additional studies performed by other groups [9, 26]. Genomic analysis of HGA and correlation of results with age and tumor site showed that H3.3G34R/V mutations characterize HGA located within the cerebral hemispheres, mainly temporo-parietal regions in adolescents and young adults with this tumor [12, 21]. K27M mutations in H3.3 and histone variant H3.1 (encoded by *HIST1H3B*) were also shown to be present in 71–78 % of diffuse intrinsic pontine gliomas (DIPGs), which are HGA of the pons [12, 24]. Moreover, *H3F3A* K27M mutations also characterize pediatric [8, 20, 21] and younger adult HGA [1] arising in the thalamus, the spinal cord or the cerebellum [8, 21] thus confirming preponderance of H3K27M mutations in HGA in the midline [12, 20, 21, 24]. These brain regions are notoriously difficult areas for surgical resection and stereotactic biopsy and the frequency and specificity of these mutations in histones strongly support their use in diagnosis and as biomarkers for these HGA tumors in children and younger patients [3, 4, 8, 22, 25].

The current routine procedure for assessing H3 status is through DNA sequencing. This is a time-consuming and laborious process which requires extraction of nucleic acids and relatively elaborate laboratory equipment that may not be available in every pathology department. In contrast, immunohistochemistry (IHC) is routinely performed in pathology laboratories. Critical insight into the effects of the K- to M change in H3 tails was provided by the Allis group who showed that this mutation acted as a gain-of-function through inhibition of the methyltransferase activity of EZH2 [14]. This leads to decreased levels of K27me3 because the Polycomb repressive complex 2 which contains EZH2 is unable to mediate this activity in the presence of H3K27M [14]. Decreased H3K27me3 levels in H3K27M-mutant HGA samples were shown by several groups further confirming this effect on tumor tissues [2, 23]. Therefore, use of decreased H3K27me3 levels by IHC was proposed as a surrogate marker to diagnose H3.3K27M mutation in the clinical setting [23]. However, the normal pattern of H3K27me3 in control brain and other brain tumors is not clearly established, and the availability of a mutation-specific antibody that specifically recognizes the mutation similar to the one derived for the most common isocitrate dehydrogenase (IDH) mutation in gliomas (IDH1R132H) would greatly help clinical management [5]. This is especially true in the context of the very poor outcome established for this specific mutation compared to H3.3 G34R/V mutations, IDH mutations, or tumors that are wild type for these genes [7, 12, 21]. We show here on a cohort of HGA enriched for midline and pediatric tumors that a commercially available rabbit polyclonal antibody directed against K27M mutant H3 variants can detect these mutations in fixed tissues using standard immunohistochemistry with high sensitivity and specificity.

## Materials and methods

### Patient samples and pathological review

All samples were obtained with informed consent after approval of the Institutional Review Board of the respective hospitals they were treated in, and were independently reviewed by pediatric neuropathologists (SA, BE, TP, AK, KL) according to the WHO guidelines. Samples were obtained from the Montreal Children’s Hospital (Montreal, McGill University Health Centre), University of Bonn (Bonn, Germany), Boston Children’s Hospital (Boston, Harvard University), Sainte-Justine Hospital (Montreal, Canada), University of Heidelberg (Heidelberg, Germany), and the Brain Tumor Toronto Bank (BTTB, Toronto). All samples were from formalin-fixed paraffin-embedded material. All midline pediatric HGA samples were from needle biopsies (pons) or partial resections (thalamus, spine, cerebellum) and most were obtained prior radio- and/or chemotherapy. Slides were available from individual tumor samples or from previously reported tissue microarrays (TMA) [6, 13, 20]. The pediatric HGA cohort (mean age 11 years) included tumor tissue from 124 WHO Grade IV astrocytomas (glioblastoma, GBM), 14 GBM recurrences, and 11 WHO Grade III astrocytomas (anaplastic astrocytoma, AA) (Table 1 **)**. A range of other brain tumors was also investigated and included other pediatric gliomas (*n* = 6), control brain (*n* = 8) and three TMAs containing, respectively, 97 medulloblastomas (MB), 71 primitive neuroectodermal tumors (PNET) samples, or 115 WHO Grade I (pilocytic astrocytomas, PA) (Supplementary Table 1). Clinical characteristics of patients with HGA are summarized in Table 1. TMAs comprised an average of three tumor cores from the same sample with a mean diameter of 1.5 mm for each core. Cores were selected from the original tumor sample and oriented on the TMA by the neuropathologist and controlled for adequate tumor representation by hematoxylin/eosin staining.

**Table 1:** Clinico-pathological characteristics and tumor genotype of high-grade astrocytomas included in this study

Diagnosis	*n*	Age median (range)	Gender	H3K27M genotype^a^
WHO grade IV astrocytomas (GBM)	118	11.0 ± 4.1	45 M, 58 F, 5 unknown	39 *H3F3A* 3 *HIST1H3B* 1 *HIST1H3C* 88 wild-type
	14 recurrences	8.7 ± 6.7		
WHO grade III astrocytomas (AA)	11	10.2 ± 2.9	1 M, 9 F,	4 *H3F3A* 1 *HIST1H3B* 6 wild-type

*GBM* glioblastoma, *AA* anaplastic astrocytoma, *M* male, *F* female

^a^Includes recurrences

### Automated immunohistochemistry

Tissue samples were cut at 5 µm, placed on SuperFrost/Plus slides (Fisher) and dried overnight at 37 °C. The slides were then loaded onto a Discovery XT Autostainer (Ventana Medical System). All solutions used for automated immunohistochemistry were from Ventana Medical System unless otherwise specified. Slides underwent de-paraffinization and heat-induced epitope retrieval (CC1 pre-diluted solution Cat# 950-124) following standard protocol. Immunostaining for H3K27M mutant and H3K27me3 were performed using a heat protocol. Briefly, rabbit polyclonal anti-H3K27M (#ABE419 Millipore, 1:500), or rabbit monoclonal anti-H3K27me3 (C36B11, #9733 Cell Signalling, diluted at 1:75) diluted in the antibody diluent (Cat# 251-018) were applied manually for 32 min at 37 °C and then incubated using the appropriate detection kit (OmniMap anti-Rabbit-HRP, Cat# 760-4311) for 8 min, followed by ChromoMap-DAB Cat# 760-159). Omission of the primary antibody was used as negative control. Slides were then counterstained with hematoxylin for 4 min, blued with Bluing Reagent for 4 min, removed from the autostainer, washed in warm soapy water, dehydrated through graded alcohols, cleared in xylene, and mounted with Permount. Sections were analyzed by conventional light microscopy. Immunohistochemistry for INI1 (clone 25/BAF47, 1:400, BD Biosciences) and CD45 (clone T29/33, pre-diluted, Dako) was performed according to the manufacturer’s instructions on a Dako autostainer. Slides were scanned using the Aperio system and independently scored for H3K27M and H3K27me3-positive staining by three independent individuals including two neuropathologists (SA, BE) blinded to the tumor genotype. Results were merged and consensus scoring was obtained as previously described [15, 20]. Briefly, samples were considered as positive for H3K27M staining if tumor cells showed nuclear staining and the core included a control blood vessel or normal brain with negative staining for the antibody. H3K27me3 staining was performed as previously described [23]. If no H3K27me3 or H3K27M staining were observed, we performed a control staining against a nuclear protein INI1 used as control for nuclear staining and tissue/fixation quality. Immunohistochemistry for INI1 (clone 25/BAF47, 1:400, BD Biosciences) and double labeling for H3K27me3 (DAB brown chromogen) and CD45 (clone T29/33, pre-diluted, Dako, AP red chromogen) was performed according to the manufacturer’s instructions on a Dako autostainer. Three HGA samples from our initial dataset were thus excluded based on negative INI1 staining. Notably, archival material fixed with Bouin’s solution showed a very strong background using the H3K27M antibody and poor H3K27me3 staining, and were therefore excluded from further analysis (*n* = 7, Supplementary Fig. 1a). Also, autopsy samples showed a high background when stained using the anti-H3K27M antibody and were not included in the dataset (Supplementary Fig. 1b).

### DNA sequencing of H3 variants

For HGA samples not previously screened using whole exome/Sanger sequencing [8, 12, 20] high-resolution melting (HRM) or pyrosequencing were used as previously described [20]. Briefly, *H3F3A* and *HIST1H3B* mutation screening using HRM was performed on a Light Cycler 480 using triplicates for each sample. Each reaction contained 10 μl of LC480 High Resolution Master Mix 2× (Roche), 0.2 μM of each primer, 2.5 μM MgCl_2_, 5–20 ng of genomic DNA and water to a final volume of 20 μl. PCR conditions were as follows: 95 °C for 10 min followed by 45–50 cycles of 95 °C for 10 s, a touchdown of 68–58 °C for 15 s (1 °C/cycle) and 72 °C for 15 s. Primers were HPLC-purified (Integrated DNA Technologies). MgCl_2_ concentration was optimized for each reaction to give low *C*_t_ values and a high plateau phase. Primer sequences are *H3F3A*-F: 5′-GTACAAAGCAGACTGCCCGCAAAT-3′, *H3F3A*-R: 5′-GTGGATACATAC AAGAGAGACTTTGTCCC-3′, and *Hist1H3B*-F: 5′-ACAGACGTCTCTGCAGGCAAGC-3′ and *Hist1H3B*-R: 5′-GGCGGTAACGGTGAGGCTTT-3′. After amplification, the PCR product was denatured at 95 °C for 1 min, cooled down to 40 °C to allow duplex formation, then high-resolution melting data were acquired when the temperature was increased from 70 to 95 °C (1 °C/cycle—25 data acquisition per °C). Analysis was performed with the Light Cycler 480 Gene scanning software. Melting curves were first normalized, which were then shifted along the temperature, and finally a difference plot was generated. The grouping method chosen was the “In-run” standards where the software applies grouping on melting standard samples included in the run.

## Results and discussion

We performed targeted sequencing using HRM or pyrosequencing to analyze the genotype of tumor and control brain samples included in this study for which information on H3.1 (*HIST31B* and *HIST31C*) and H3.3 (*H3F3A*) mutational status at position K27 was not available. We identified a K27M substitution in 48 samples, 5 HGA carrying H3.1K27M (4 on *HIST1B* and 1 on *HIST1C*) and 43 HGA with H3.3K27M. Six HGA cases had no available material to perform genotype analysis and were excluded from further analysis.

**Fig. 1:**
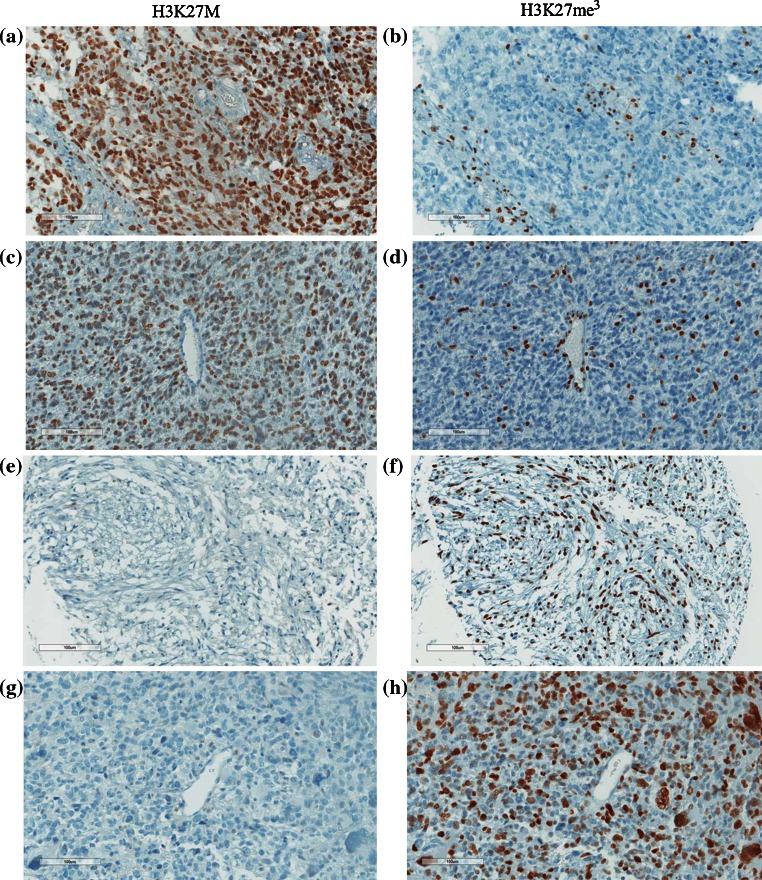
Immunohistochemical (IHC) staining of pediatric high-grade astrocytomas (HGA) using the anti-H3K27M antibody correlates with tumor genotype and decreased H3K27Me3 in tumors. Representative IHC of pediatric HGA using anti-H3K27M (**a**, **c**, **e**, **g**) or anti-H3K27me3 (**b**, **d**, **f**, **h**) antibodies and counterstained with hematoxylin. H3K27M shows strong nuclear positivity in tumor cells, but no staining in the nuclei of endothelial and smooth muscle cells in blood vessels in K27M mutant tumors (**a**, **c**). Tumors wild type for H3K27 show no nuclear staining with the anti-H3K27M antibody (**e**, **g**). Corresponding H3K27me3 staining on the same samples shows global decrease of the expression of this histone mark in H3K27M mutant tumors (**b**, **d**) compared to tumors wild type for this mutation (**f**, **h**). Notably, positivity for H3K27me3 was mainly seen in tumor vessels (**b**, **d**) even though a degree of intra-tumor staining was also seen in H3K27M mutant samples (**d**)

**Fig. 2:**
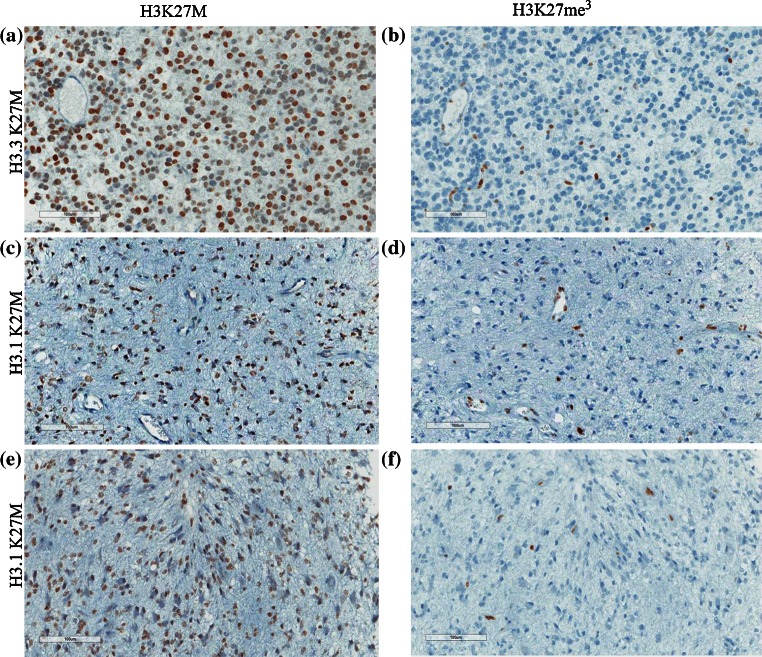
Immunohistochemical (IHC) staining of H3.1K27M and H3.3K27M mutant high-grade astrocytomas (HGA) shows similar results using the anti-H3K27M and anti-H3K27me3 antibodies. Representative sections from HGAs (WHO Grade III) carrying H3.3K27M (**a**, **b**) and H3.1K27M (**c**, **d**, **e**, **f**) immunostained with anti-H3K27M (**a**, **c**, **e**) and anti-H3K27me3 (**b**, **d**, **f**) antibodies. Two areas from the same H3.1K27M WHO Grade III DIPG sample are shown to further illustrate that the K27M mutation is also present in seemingly lower grade tumor sections (**c**)

We then assessed H3K27M expression by IHC on the 143 genotyped HGA (118 GBM, 14 GBM recurrences, 11 AA, Table 1). Results were compared to H3K27me3 staining on consecutive sections and to genomic data. Anti-H3K27 M antibody showed a strong nuclear staining of most tumor cells (>80 %) in all 48 samples with the H3K27M genotype (Fig. 1; Tables 1, 2). None of the H3K27M wild-type samples showed nuclear staining (Table 2; Fig. 1; Supplementary Fig. 2). Importantly, the antibody was able to recognize both H3.3 and H3.1 K27M mutant tumors (Fig. 2), as expected given that the peptide it was raised against is identical in both mutant H3 variants. The anti-H3K27M antibody did not stain any of the H3.3G34R/V HGA included in this dataset (*n* = 7). It did not recognize endothelial cells or other vascular structures within H3K27M mutant HGA which still expressed H3K27me3 (Fig. 1; Supplementary Fig. 2).

**Table 2:** Comparison between immunohistochemical analysis of H3K27M mutations and tumor genotype in high-grade astrocytomas included in this study

Genotype	Positive H3K27M staining	Negative H3K27M staining
H3K27 wild type	0	84^a^
H3K27M	48^a^	0

^a^Includes recurrences (3 H3K27M mutants and 11 wild types)

H3K27me3 staining was negative or strongly decreased in K27M mutant samples as previously described [2, 14, 23], while tumor cells in samples wild type for H3K27M showed high expression of this post-translational histone modification (Fig. 1). Out of the 14 recurrent GBM, 3 were genotyped to be H3K27M mutants. All three GBM showed strong H3K27M expression across most tumor cells. Conversely, recurrent samples wild type for this mutation showed no staining. This further confirms that when H3K27M is identified at diagnosis it is maintained by most tumor cells at relapse and is not acquired at the time of tumor recurrence similar to IDH-mutant gliomas [10]. To further assess the specificity of H3K27M antibody, we screened TMAs containing PA, MB and PNET samples and a set of other pediatric brain tumors or normal brain (Supplementary Table 1). As expected, none of these samples had positive H3K27M staining (Supplementary Table 1, Supplementary Fig. 3 and data not shown).

**Fig. 3:**
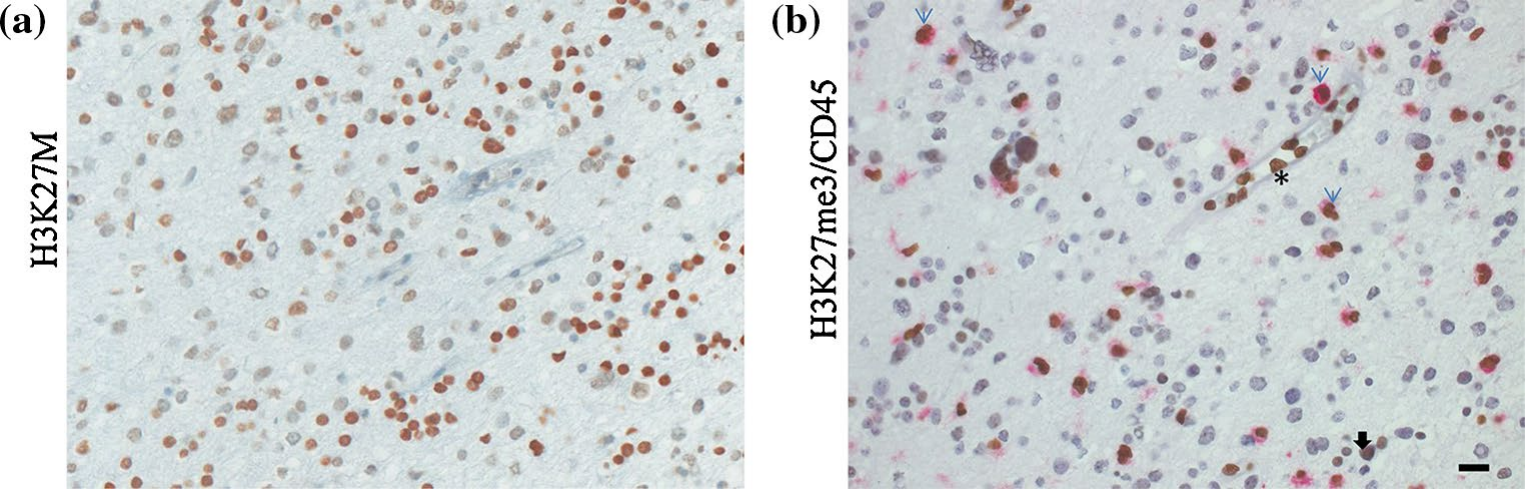
H3K27M staining and H3K27me3/CD45 co-immunostaining on consecutive slides of an H3K27M mutant high-grade astrocytoma. **a** anti-H3K27M staining (nuclear); **b** co-immunostaining using a CD45 antibody (cytoplasmic, *purple*) and an H3K27me3 antibody (nuclear, *dark brown*); results indicate that only a proportion of cells positive for H3K27me3 staining in H3K27M mutant high-grade astrocytomas are positive for CD45 staining and represent a lymphocytic infiltrate (*light blue arrows*). Endothelial and smooth muscle vascular cells show uptake of the H3K27me3 antibody alone (*asterisk*) while few cells within the tumor remain positive for H3K27me3 (*black arrow*)

A small number of samples (*n* = 6) wild type for H3K27M showed scattered cells with pale, nonspecific-appearing cytoplasmic staining with the anti-H3K27M antibody, mostly in areas of necrosis or inflammation, but these cases were not viewed as equivocal and were not interpreted as positive by scoring pathologists blinded to the genotype. As for the anti-H3K27me3 antibody, some strong extravascular nuclear staining was seen in ~10 % of H3K27M mutant samples (which generally do not stain except in blood vessels). In some cases the staining appeared mainly to be from mononuclear inflammatory cells including microglia, which we confirmed by immunostaining and co-immunostaining with anti-CD45, a marker that recognizes lymphocytes, on consecutive slides from the same tumors (Fig. 3). However, nuclear staining for H3K27me3 in H3K27M samples could not be solely accounted for by CD45-positive cells, which highlights the fact that loss of H3K27me3 is not reliable to predict H3K27M genotype (Fig. 3b; Supplementary Fig. 4). In addition, based on the limits of IHC at the single cell level, we cannot exclude that a small subset of tumor cells is negative for both H3K27M and H3K27me3 in H3K27M tumor samples (Supplementary Fig. 4).

Our results indicate that the rabbit monoclonal antibody directed against H3K27M mutations specifically recognizes mutant proteins using simple IHC. Nuclear immunostaining was restricted to tumor cells in samples carrying the mutant H3K27M genotype, both in H3.1 and H3.3. This staining is more specific than H3K27me3 investigation as it specifically targets the mutation and not one of its downstream effects. Other pediatric posterior fossa tumors including ependymomas [16] and different sub-groups of medulloblastomas [11, 17–19] have strong H3K27me3 positive staining based on their own biology. In addition, based on the scattered H3K27me3-positive staining in several mutant H3K27M HGA, interpretation of the results may prove challenging when using H3K27me3 expression levels as a surrogate marker of H3K27M. In summary, our data show that the H3K27M antibody is highly useful for tumor diagnosis in the clinical setting where IHC is routine procedure in pathology laboratories. This is especially true in the context of midline and hindbrain tumors where surgical material is limited. The concurrent use of this antibody with other known prognostic pediatric HGA markers including TP53 will help orient patients to optimal clinical care and management based on the very poor prognosis and limited survival seen in the context of this mutation.

## Electronic supplementary material

Below is the link to the electronic supplementary material.

Supplementary material 1 (PDF 6 kb)

Supplementary material 2 (PDF 638 kb)
