# Supplementary material for: Specific detection of methionine 27 mutation in histone 3 variants (H3K27M) in fixed tissue from high-grade astrocytomas
Source: Acta Neuropathol. 2014 Sep 9;128(5):733–41. doi: 10.1007/s00401-014-1337-4 (PMC4201745; doi:10.1007/s00401-014-1337-4)
Supplement: Supplementary file 1 — Supplementary material 1 (PDF 6 kb) [file 401_2014_1337_MOESM1_ESM.pdf]

Supplementary Table 1: Other primary brain tumors included in this study with a known wild-type genotype for H3K27

Tumor type	n
Medulloblastoma	99
Primitive Neuro-ectodermal Tumor	71
Pilomyxoid astrocytoma	1
Ependymoma	2
Pilocytic astrocytoma<br>(WHO Grade I Astrocytomas)	115
WHO Grade II Astrocytoma	3
