# Supplementary material for: Specific detection of methionine 27 mutation in histone 3 variants (H3K27M) in fixed tissue from high-grade astrocytomas
Source: Acta Neuropathol. 2014 Sep 9;128(5):733–41. doi: 10.1007/s00401-014-1337-4 (PMC4201745; doi:10.1007/s00401-014-1337-4)
Supplement: Supplementary file 2 — Supplementary material 2 (PDF 638 kb) [file 401_2014_1337_MOESM2_ESM.pdf]

(a) (b)

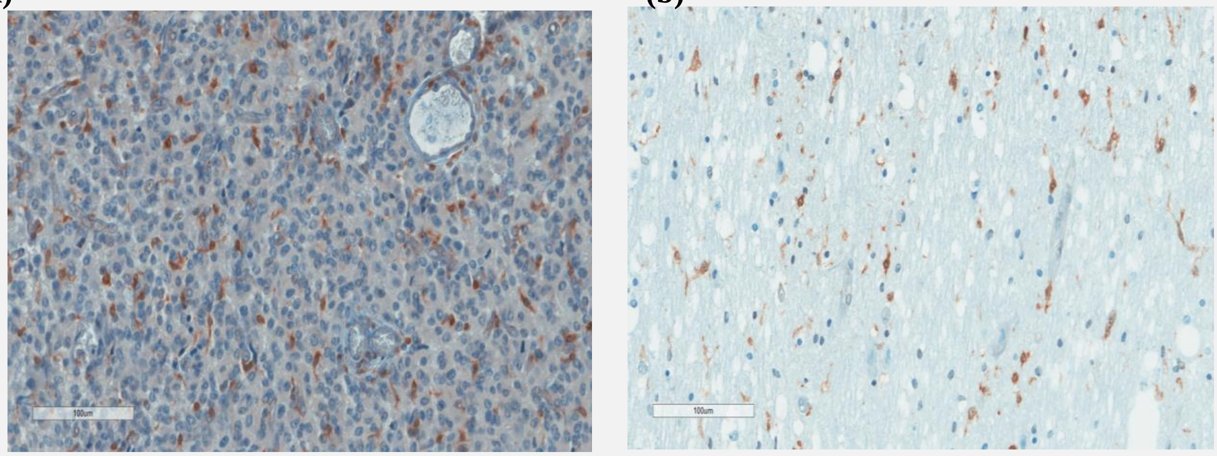

Supplementary Figure 1: Representative immunohistochemistry of H3K27M mutant pediatric WHO Grade IV astrocytomas with H3K27M antibody on slides fixed using Bouin solution (a) or an autopsy case (b). Note the stronger background with non-specific cytoplasmic staining in both cases.

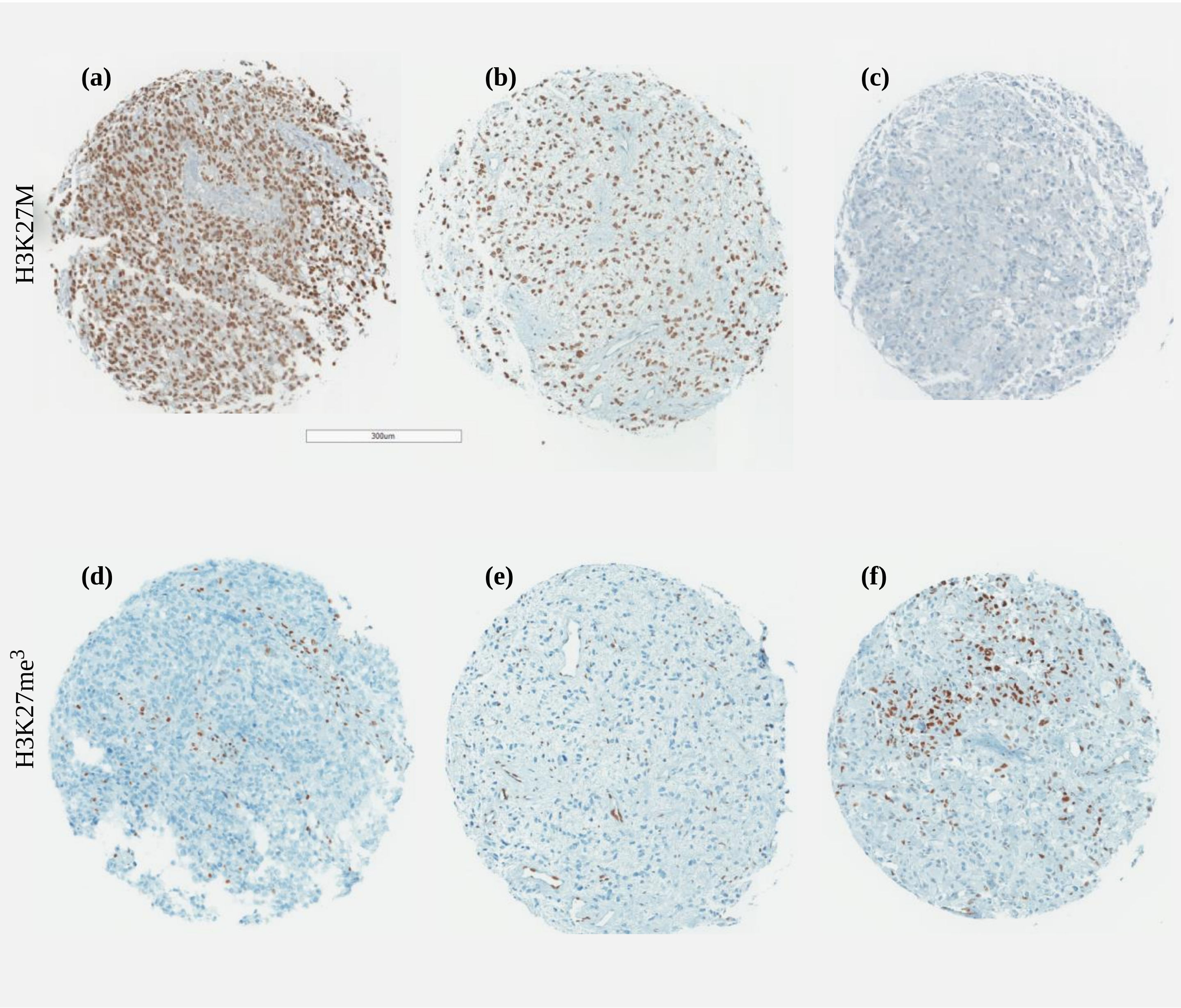

Supplementary Figure 2. Representative immunohistochemistry of pediatric WHO Grade IV astrocytomas with H3K27M antibody (a, b, c) and H3 trimethyl-K27 antibody (d, e, f) and counterstaining with hematoxylin. K27M mutant tumors show strong nuclear staining for K27M in tumor cells but no staining in vessels (a) and (b). A wild-type K27 tumor (c) shows no nuclear staining for K27M. In the mutant tumors, staining for H3 trimethyl-K27 is confined to vessels (d, e) while it is more widespread in the wild-type tumor (f).

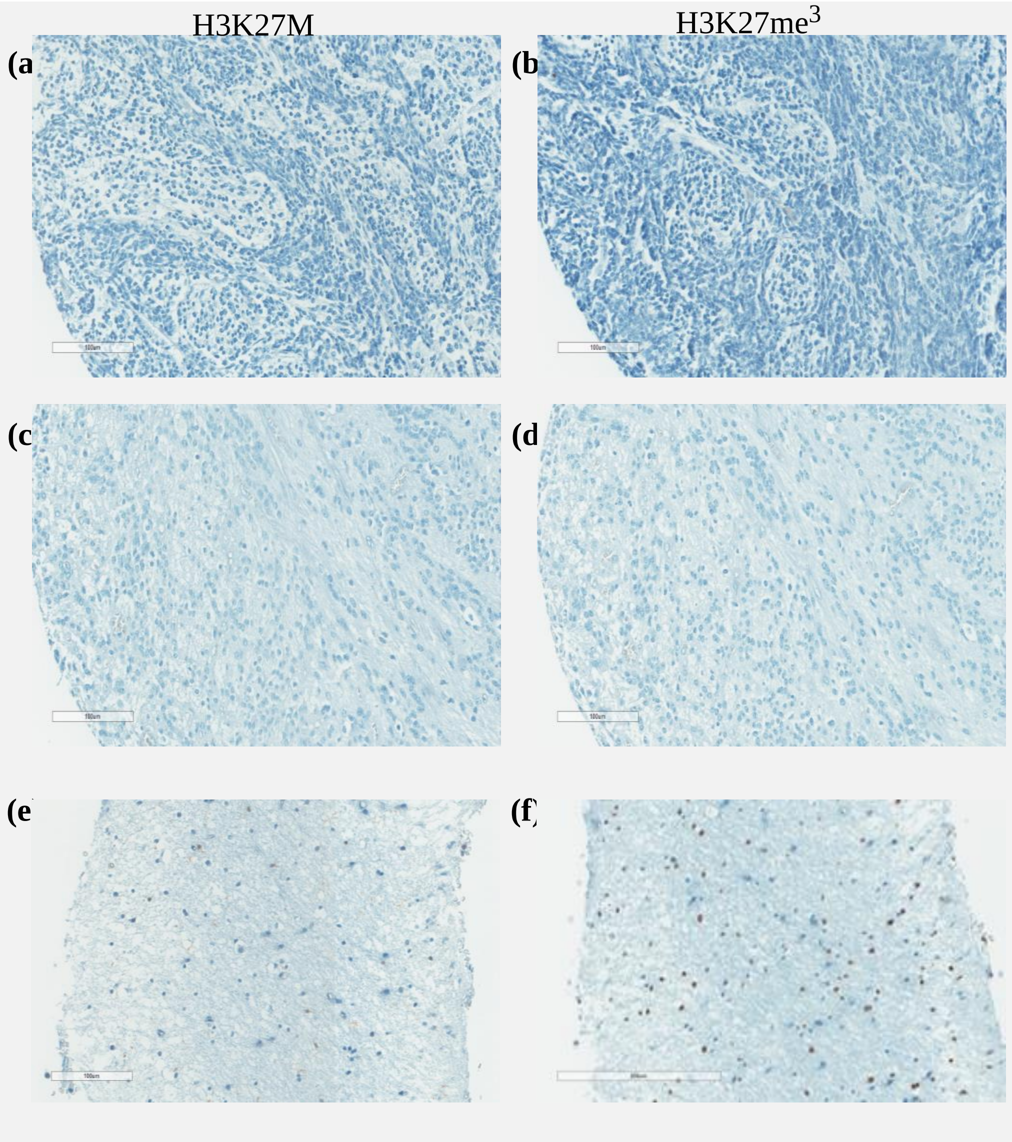

Supplementary Figure 3. Representative images of immunohistochemistry with antibodies against H3K27M and H3 trimethyl-K27 and counterstaining with hematoxylin in a medulloblastoma (a, b), a Primitive Neuro-Ectodermal Tumor (c, d) and a WHO Grade I (pilocytic) astrocytoma (e, f). The tumors on the respective TMAs are negative for H3K27M.

(a) (b)

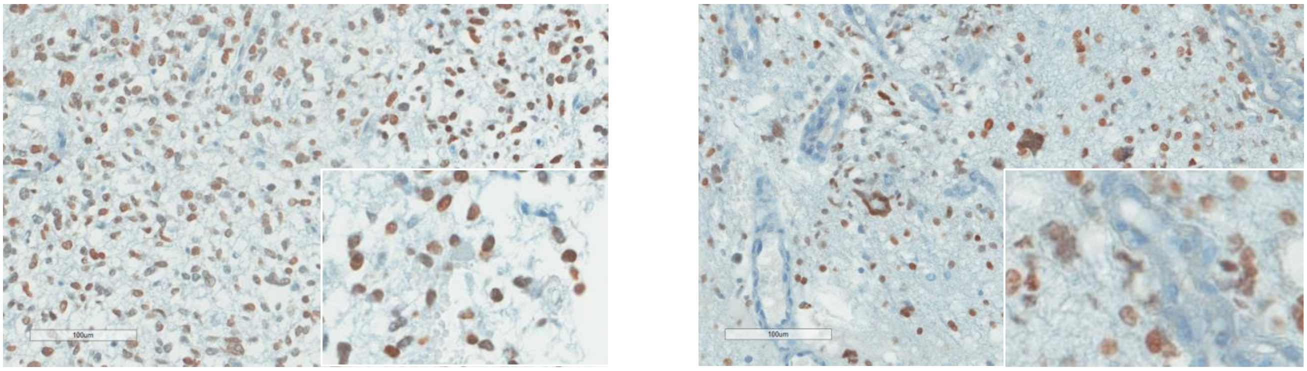

Supplementary Figure 4 Variability of the IHC staining of K27M mutant pGBM with H3K27M antibody and counterstaining with hematoxylin (a, b). This may reflect true differences in the level of expression or technical differences related to duration of fixation etc. In sections from few samples, tumor cells will show no uptake of the antibody (a) similar to vascular structures (b).
